# Social network analysis and whole-genome sequencing to evaluate disease transmission in a large, dynamic population: A study of avian mycobacteriosis in zoo birds

**DOI:** 10.1371/journal.pone.0252152

**Published:** 2021-06-09

**Authors:** Carmel Witte, James H. Fowler, Wayne Pfeiffer, Laura L. Hungerford, Josephine Braun, Jennifer Burchell, Rebecca Papendick, Bruce A. Rideout

**Affiliations:** 1 Disease Investigations, San Diego Zoo Wildlife Alliance, San Diego, California, United States of America; 2 Department of Family Medicine and Public Health, University of California, San Diego, La Jolla, California, United States of America; 3 Graduate School of Public Health, San Diego State University, San Diego, California, United States of America; 4 Department of Political Science, University of California, San Diego, La Jolla, California, United States of America; 5 San Diego Supercomputer Center, University of California, San Diego, La Jolla, California, United States of America; 6 Department of Population Health Sciences, Virginia-Maryland College of Veterinary Medicine, Blacksburg, Virginia, United States of America; Cambridge University, UNITED KINGDOM

## Abstract

This study combined a social network analysis and whole-genome sequencing (WGS) to test for general patterns of contagious spread of a mycobacterial infection for which pathways of disease acquisition are not well understood. Our population included 275 cases diagnosed with avian mycobacteriosis that were nested in a source population of 16,430 birds at San Diego Zoo Wildlife Alliance facilities from 1992 through mid-2014. Mycobacteria species were determined using conventional methods and whole genome sequencing (WGS). *Mycobacterium avium avium* (MAA) and *Mycobacterium genavense* were the most common species of mycobacteria identified and were present in different proportions across bird taxa. A social network for the birds was constructed from the source population to identify directly and indirectly connected cases during time periods relevant to disease transmission. Associations between network connectivity and genetic similarity of mycobacteria (as determined by clusters of genotypes separated by few single nucleotide polymorphisms, or SNPs) were then evaluated in observed and randomly generated network permutations. Findings showed that some genotypes clustered along pathways of bird connectivity, while others were dispersed throughout the network. The proportion of directly connected birds having a similar mycobacterial genotype was 0.36 and significant (p<0.05). This proportion was higher (0.58) and significant for MAA but not for *M*. *genavense*. Evaluations of SNP distributions also showed genotypes of MAA were more related in connected birds than expected by chance; however, no significant patterns of genetic relatedness were identified for *M*. *genavense*, although data were sparse. Integrating the WGS analysis of mycobacteria with a social network analysis of their host birds revealed significant genetic clustering along pathways of connectivity, namely for MAA. These findings are consistent with a contagious process occurring in some, but not all, case clusters.

## Introduction

Social network analysis coupled with traditional epidemiologic contact tracing and whole genome sequencing (WGS) can refine our understanding of disease epidemiology. The social network provides important visualization and captures contact heterogeneity, while genetic data provide the resolution to identify true transmission pathways [1, 2]. These methods for investigating disease epidemiology can be especially useful for diseases that are not well understood due to more than one transmission pathway, multiple causal pathogens, complex contact patterns, and long and variable incubation times. The approach has been used to elucidate transmission pathways for *Mycobacterium tuberculosis* [3, 4] and *Mycobacterium abscessus* [5] in humans and *Mycobacterium bovis* in wildlife [6].

The goal of the current study was to use social network analysis and WGS to identify complex disease transmission patterns using avian mycobacteriosis as a model. The epidemiology of avian mycobacteriosis is not well understood. This chronic disease of birds with an insidious onset and variable incubation time is generally considered to be contagious via the fecal-oral route [7]. However, studies both historic (reviewed by Feldman [8]) and recent [9–11] support only low bird-to-bird transmissibility. Other studies [12–15] have found diverse mycobacteria from clusters of cases, suggesting that the infections could not have arisen from the same source. Environmental sources such as soil or water may also be sources of infection [16], similar to non-tuberculous mycobacterial infectious in humans and related mycobacteria in other animals [17–19]. Combining social network analysis with WGS can improve understanding of these transmission pathways.

In a previous study [20], we used WGS to characterize mycobacteria in birds from the San Diego Zoo and Safari Park (collectively referred to as San Diego Zoo Wildlife Alliance: SDZWA). We found high diversity between individual isolates but also groups of closely related genotypes. Inferring transmission from WGS data alone was not possible because of incomplete sampling and lack of information on complex temporal contact patterns between birds.

In a second study [11], we evaluated general patterns of disease spread by examining direct and indirect connectivity of cases, using spatial and temporal variation in the social network structure to isolate patterns attributed to contagion. Cases of mycobacteriosis were significantly clustered in a way that was highly suggestive of a contagious process. However, we could not distinguish between clusters arising from similar versus genetically diverse mycobacteria.

Herein, we combine social network analysis and WGS to investigate patterns of mycobacterial disease in birds from a complete network with 25 years of follow-up and near-complete case ascertainment. Specifically, we use the combined network and genetic data to test for evidence of contagious spread through the contact network. Findings from this study provide additional insight to the complex epidemiology of avian mycobacteriosis.

## Materials and methods

### Source population

The source population included 16,867 birds present at SDZWA between 1 January 1992 and 1 June 2014. This included all birds that were six months old or older and living within SDZWA facilities for at least 7 days during the study period. Birds in this source population were under continual health monitoring by keepers and veterinary staff throughout the study period and received post-mortem exams if they died. The population was dynamic, with birds being imported, exported, and moved between enclosures for breeding or other management reasons. This housing history was tracked electronically over time and included individual-level information on the specific enclosure and when each bird moved in and out. These enclosure moves captured potential exposure to other birds infected with avian mycobacteriosis. Enclosure-sharing could not be determined from housing history records for 437 of the birds, so these birds were removed from the study. The final population of 16,430 birds, representing 950 species and subspecies, was used to identify all birds diagnosed with avian mycobacteriosis and create a social network to link connected cases. Detailed data on this source population and the derived social network have been published [11].

All data in these retrospective analyses were originally collected for medical activities and animal management purposes unrelated to the present study. For these reasons, the San Diego Zoo Global Institutional Animal Care and Use Committee exempted our study from review for the ethical use of animals in research.

### Case identification

In this source population, 275 birds were diagnosed with avian mycobacteriosis [11]. When a bird from the source population died, a board-certified veterinary pathologist conducted a post-mortem exam that included histopathology on complete sets of tissues unless advanced autolysis precluded evaluation. If lesions suggestive of mycobacterial disease were observed, then Ziehl-Neelsen or Fite-Faraco special stains were used to confirm the presence of acid-fast-bacilli. Any bird with acid-fast bacilli present in tissues was considered positive for avian mycobacteriosis. Most cases were identified post-mortem, but occasionally clinical presentation permitted diagnosis from a biopsy.

### Network construction

Among the 275 cases, 203 birds were identified as the study “subjects”, i.e., the subset of birds that either hatched at SDZWA or were imported and observed in the population for at least two years (a presumed maximum incubation time). A network was then constructed that linked subjects to other birds in the source population that they shared an enclosure with, i.e., their “friends”. Each subject could have multiple friends because they were housed with multiple birds during the study dates; a bird that was a subject could also serve as a friend for another subject.

The network was assembled from the entire source population in the same manner as previously described [11], defining connectivity between subjects and friends when two birds shared an enclosure for at least 7 days during the subject’s plausible incubation time and its friend’s infectious period. The subject’s incubation time was assumed to be between 6 and 24 months before the subject’s date of diagnosis. The minimum incubation time is consistent with early experimental transmission studies in birds [21, 22] and with our own observations of the earliest case occurring at 182 days of age [9]. As for the maximum incubation time, early experimental studies report deaths from avian mycobacteriosis 12–14 months after infection [21–23]; however, some experts believe it could take years for a bird to succumb to the disease [8]. No information was available for plausible time periods when a bird may shed mycobacteria. Therefore, the friends’ infectious times were set to the maximum incubation time of 24 months prior to the friends’ final date in the study, which corresponded to death dates, removal dates, or the end of the study. Importantly, the entire population of 16,430 birds was used to link case subjects to their directly connected friends, and their indirectly connected “friends of friends”. Birds that were not cases were then removed to retain a network of just the subset of cases and their epidemiologic links over time.

### Determination of mycobacteria genetic relatedness

Isolation and species determination of mycobacteria from infected birds were attempted for 167 of the 275 cases of mycobacteriosis. Reasons for not attempting culture were unrelated to this study, and included lack of available tissues (e.g., lesions not present in available tissues, tissues discarded, advanced autolysis) or culture was not of clinical or prognostic value for that case. Fresh or frozen tissues (other than feces) were collected using aseptic techniques and submitted to either the Molecular Diagnostics Laboratory (San Diego Zoo Global, Escondido, CA) or an external microbiology laboratory (University of California San Diego Health System Clinical Laboratory, La Jolla, CA; National Jewish Health Advanced Diagnostic Laboratories, Denver, CO; National Veterinary Services Laboratory, Ames, IA; or University of Wisconsin, School of Veterinary Medicine Mycobacteriology Laboratory, Madison, WI) for mycobacterial culture and species determination using DNA probes, HPLC, or Sanger sequencing.

DNA was extracted from isolates that were viable at the time of the study using QIAamp DNA Mini Kit (Qiagen, Valencia, CA) following the manufacturers protocol with the pretreatment steps previously described [20]. When at least 0.3 μg of DNA could be extracted, the sample was sent to The Scripps Research Institute Next Generation Sequencing Core (La Jolla, CA) for WGS on a HiSeq 2000 or a NextSeq 500 (Illumina, La Jolla, California). Sequencer reads for isolates confirmed to contain mycobacteria were deposited in the NCBI Sequence Read Archive under Bioproject PRJNA351843.

The subsequent genomic analysis has been described in detail [19]. In summary: (1) reads for each isolate were assembled into contigs with Velvet 1.2.10 [24]; (2) contigs were aligned against the NCBI RefSeq database using BLAST+ 2.2.29 [25] after which custom scripts identified the bacterial species and strains; (3) variants between isolates of the same species were called using the GATK 3.5 HaplotypeCaller tool [26] and auxiliary tools; (4) custom scripts were used to retain only the high-confidence single nucleotide polymorphisms (SNPs), to handle isolates that contain more than one genotype (i.e., SNP sequence), to align the genotypes within the same species, and to compute the genetic distance in number of SNPs between each pair of genotypes; (5) clusters of closely related genotypes were identified from phylogenetic trees generated using RAxML 8.2.9 [27] and visualized with FigTree 1.4.0 (http://tree.bio.ed.ac.uk/software/figtree). Recent refinements to the scripts in Step 4 allowed us to identify more high-confidence SNPs in *M*. *genavense* isolates and better handle those with multiple genotypes.

Genotypes between directly and indirectly connected cases were characterized as “similar” (i.e., likely part of the same transmission chain) if they were within 12 SNPs of at least one other genotype in a genomic cluster. The threshold value of 12 SNPs was used as an indication of the maximum possible genetic diversity within and between hosts as previously defined for *M*. *tuberculosis* [28, 29]. If genomic data were not available for both of the birds, then the network edge was classified as “unknown”. While including a transmission threshold allowed us to test our hypotheses and interpret findings, we acknowledge that there are limitations to this assumption (see Discussion). To address the 12-SNP-threshold assumption, we also removed the cutoff and evaluated the relatedness of mycobacteria based on continuous distributions of pairwise SNPs between connected (and non-connected) birds as a measure of the relatedness of mycobacteria. This latter method was restricted to comparing MAA and *M*. *genavense* sequences, separately.

### Statistical and network analyses

Mycobacterial species identified from the 275 cases were summarized by isolation method, and taxa of the host bird. To characterize the tendency for certain avian taxa to become infected with the different species of mycobacteria, we compared proportions of birds from different taxonomic orders that were infected with MAA (versus all other known mycobacteria species) with Fisher’s exact tests. The same tests for proportionality were also applied to birds infected with *M*. *genavense*. All comparisons were limited to avian taxonomic orders where at least ten birds had mycobacteria species identified.

Network visualizations and analyses were performed in R software (package: igraph [30]). The network of 275 cases was graphed using the Fruchterman-Reingold algorithm [31] to illustrate connectivity between directly and indirectly connected cases and to visualize prevalent genotype groups. The algorithm is a visualization tool that optimizes placing connected nodes close to each other and unconnected nodes far from each other. Two different node centrality measures were evaluated based on node connectivity within the larger source population: degree centrality (the number of connected nodes) and eigenvector centrality (the extent to which a node is connected to other highly connected nodes) [32]. Distributions of these two measures were compared between birds with MAA and *M*. *genavense*, as well as between those with known and unknown genotypes according to the Kolmogorov-Smirnov test for equality of distributions. The median number of days that connected cases with MAA and *M*. *genavense* spent together were determined. Differences in days spent together for cases that shared versus those that did not share a similar genotype were evaluated with a Mann-Whitney U test.

The proportion of connected cases having similar genotypes was determined by dividing the number of subject-friend pairs with similar genotypes by the total number of connected pairs with known genotypes for both birds. This proportion was then compared to the distribution of the same calculation on 1,000 randomly generated null networks where the network topology and prevalence of each genotype was preserved, but the genotypes (including unknown genotypes) were randomly shuffled to different nodes using methods previously described [11, 33, 34]. If the observed proportion was outside the range of the 2.5^th^ and 97.5^th^ percentiles of the null distribution (i.e., the null 95% confidence interval), then the null hypothesis that the observed proportion could have arisen from chance was rejected. Reported p-values were estimated from the null 95% CI. The calculation was performed for all directly and indirectly connected cases, as well as among those with just MAA or just *M*. *genavense*. Of note, evaluations for indirectly connected cases were limited to the pairs where the friend of friend lived in a different aviary than the subject but could have influenced the disease outcome in the subject based on the timing of contact with another mutual friend. This ‘friends of friends’ method [11] was used to isolate and test for patterns of contagion within the network structure, as previously reported [11].

To determine whether genotypes of connected cases were more related than genotypes of non-connected cases, the numbers of SNPs between pairs of birds with MAA and *M*. *genavense* were summarized separately for subject-friend pairs with WGS. The observed distribution of SNPs was then compared to the distribution generated from 1,000 random permutations as described above. Significance was determined with the Kolmogorov-Smirnov test for equality of distributions. This was repeated using genetic relatedness between indirectly connected cases.

## Results

### Bird population and mycobacteria summaries

The 275 cases of avian mycobacteriosis represented 149 species of birds. On average, 12 cases were diagnosed per year (SD = 5; range 4–20). The median time spent in the population was 4.5 years (1,638 days; interquartile range or IQR: 2–8.2 years; range: 33 days-26 years). Excluding quarantine and hospitalization-related moves, cases moved on average 5 times during the study period (SD = 4.5) and were associated, on average, with 3.5 different enclosures (total represented enclosures among cases = 377).

#### MAA and M. genavense were the most common species of mycobacteria

Species of mycobacteria were determined for 124/275 of the infected birds (45%; Table 1). *Mycobacterium avium avium* (MAA) was most commonly identified (52/124; 42%) but was also the most frequently tested for as culture methods were not optimized for *Mycobacterium genavense* during the early part of the study period. *M*. *genavense* was identified in 44 birds (out of 124; 35%). Of the birds with WGS data, the numbers with MAA and *M*. *genavense* were similar (n = 37 and n = 41, respectively). Eleven cases with WGS (out of 97; 11%) were *M*. *a*. *hominissuis*. Nine additional species or subspecies of Mycobacterium (or Mycolicibacter or Mycolicibacterium) were identified; isolates from five birds were identified to the *M*. *avium* complex level, and one was identified as a rapid grower (Table 1).

**Table 1:** Species of Mycobacterium identified in 124 out of 275 infected birds at San Diego Zoo and Safari Park, 1992–2014.

Species of mycobacteria and related genera	Number of birds with mycobacterial species determination by:		Total
	WGS^a^	Other method^b^	
*M*. *avium avium*			
MAA	37	14	51
MAA & *M*. *xenopi*		1	1
*M*. *avium hominissuis*	11		11
*M*. *genavense*			
*M*. *genavense*	40	3	43
*M*. *genavense* & *M*. *intracellulare*	1		1
Other species			
*Mycolicibacter arupense*	1		1
*Mycolicibacterium fortuitum*	2		2
*Mycolicibacterium hassiacum* & *Mycolicibacterium peregrinum*	1		1
*M*. *intracellulare*	2	2	4
*Mycolicibacterium porcinum*	1		1
*M*. *xenopi*		1	1
URHD0025	1		1
*M*. *avium* complex (not further identified)		5	5
rapid grower (not further identified)		1	1
Totals	97	27	124

MAA = *Mycobacterium avium avium*; WGS = Whole genome sequence

^a^Subset of those previously reported in Pfeiffer et al. 2017 [20]; *Mycolicibacterium porcinum* was previously reported as *Mycobacterium vulneris*; the updated nomenclature reflects recent taxonomy revisions.

^b^Other methods used for species determination included Sanger sequencing, DNA probe, high performance liquid chromatography (HPLC), and partial genome sequencing.

For the cases in the present study, 115 distinct WGS from 97 birds (35% of 275) were obtained. This included 15 groups of genetically similar mycobacteria containing 2 or more birds (7 separate groups of MAA, representing 25 birds; 7 separate groups of *M*. *genavense*, representing 31 birds; 1 group of *M*. *a*. *hominissuis*, representing 2 birds). Many birds (n = 39) had sequences far apart from all other isolates, including seven additional species of mycobacteria (shown in Table 1) as well as distinct isolates of MAA, *M*. *a*. *hominissuis*, and *M*. *genavense* [20].

#### Proclivity for infection by MAA or *M*. *genavense* differed across avian taxa

Species of mycobacteria are summarized by avian taxonomic orders in Table 2. The number of mycobacteriosis cases observed across the different avian taxa likely reflects the proportion of those birds in the SDZWA population, with Anseriformes (waterfowl), Passeriformes (perching birds), and Columbiformes (pigeons and doves) being the most common avian taxa in this population [9]. However, comparisons of relative proportions show that birds in certain taxa tend to acquire infection from MAA and others tend to acquire infection from *M*. *genavense*. Among taxa with enough cases for statistical evaluation, MAA was more common in Anseriformes (90%; 28/31), followed by Columbiformes (34%; 12/35) and Passeriformes (8%; 2/24), and these proportions were statically different from each other (p<0.05 for all comparisons; Table 2). Likewise, *M*. *genavense* was most common in Passeriformes (79%; 19/24) followed by Columbiformes (46%; 16/35; p = 0.02), with no cases identified in Anseriformes; these proportions were also significantly different (Table 2). Our findings assumed that the unknown/missing data on mycobacterial species do not bias the results.

**Table 2:** Summary of avian taxa and species of Mycobacterium (and related genera) identified from 275 cases of avian mycobacteriosis at San Diego Zoo and Safari Park, 1992–2014.

Avian taxonomic order (no. bird species represented)	MAA	*M*. *genavense*	*M*.*a*.*hominissuis*	Other *M*. *spp*.^a^ and related genera	Unknown spp.	Total Cases	% MAA (MAA / known *M*. *spp*)	% *M*. *genavense (M*. *genavense /* known *M*. *spp*.*)*
Anseriformes (23)	28			3	10	41	90% (28/31)^b^	0% (0/31)^c^
Bucerotiformes (3)				2^d^	4	6		
Charadriiformes (2)		1			2	3		
Ciconiiformes (1)		2			1	3		
Colliformes (1)		1^d^			2	3		
Columbiformes (31)	12	16	1	6	30	65	34% (12/35)^b^	46% (16/35)^c^
Coraciiformes (6)	1	1	1	1	5	9		
Galliformes (9)	2		5	1	15	23		
Gruiformes (1)	1^d^					1		
Musophagiformes (3)		1			2	3		
Otidiformes (1)	2					2		
Passeriformes (49)	2	19	2	1	63	87	8% (2/24)^b^	79% (19/24)^c^
Phoenicopteriformes (2)	1			1		2		
Piciformes (3)	1	1		1	1	4		
Psittaciformes (12)	2	2	2	1	14	21		
Strigiformes (1)					1	1		
Struthioniformes (1)					1	1		
Totals:	52	44	11	17	151	275		

MAA = *Mycobacterium avium avium*

^a^Other identified species of Mycobacterium are listed in Table 1.

^b^Avian taxonomic orders with the same letter indicate a statistically significant difference in proportions of birds with MAA (Fisher’s exact p-value <0.001 for all comparisons, except Columbiformes versus Passeriformes, where p = 0.04). Comparisons were limited to taxa where at least ten birds had known species of Mycobacterium.

^c^Avian taxonomic orders with the same letter indicate a statistically significant difference in proportions of birds with *M*. *genavense* (Fisher’s exact p-value <0.001 for all comparisons, except Passeriformes versus Columbiformes, where p = 0.02). Comparisons were limited to taxa where at least ten birds had known species of Mycobacterium.

^d^Two different species of Mycobacterium (or Mycolicibacterium) were isolated from the same bird: *M*. *hassiacum* and *M*. *peregrinum* were identified in a black-casqued hornbill (*Ceratogymna atrata*) by WGS; *M*. *genavense* and *M*. *intracellulare* were identified in a Blue-naped mousebird (*Urocolius macrourus*) by WGS; MAA and possibly *M*. *xenopi* were identified in an East African gray-crowned crane (*Balearica regulorum*) by DNA probe.

### Social network summaries

**Fig 1:**
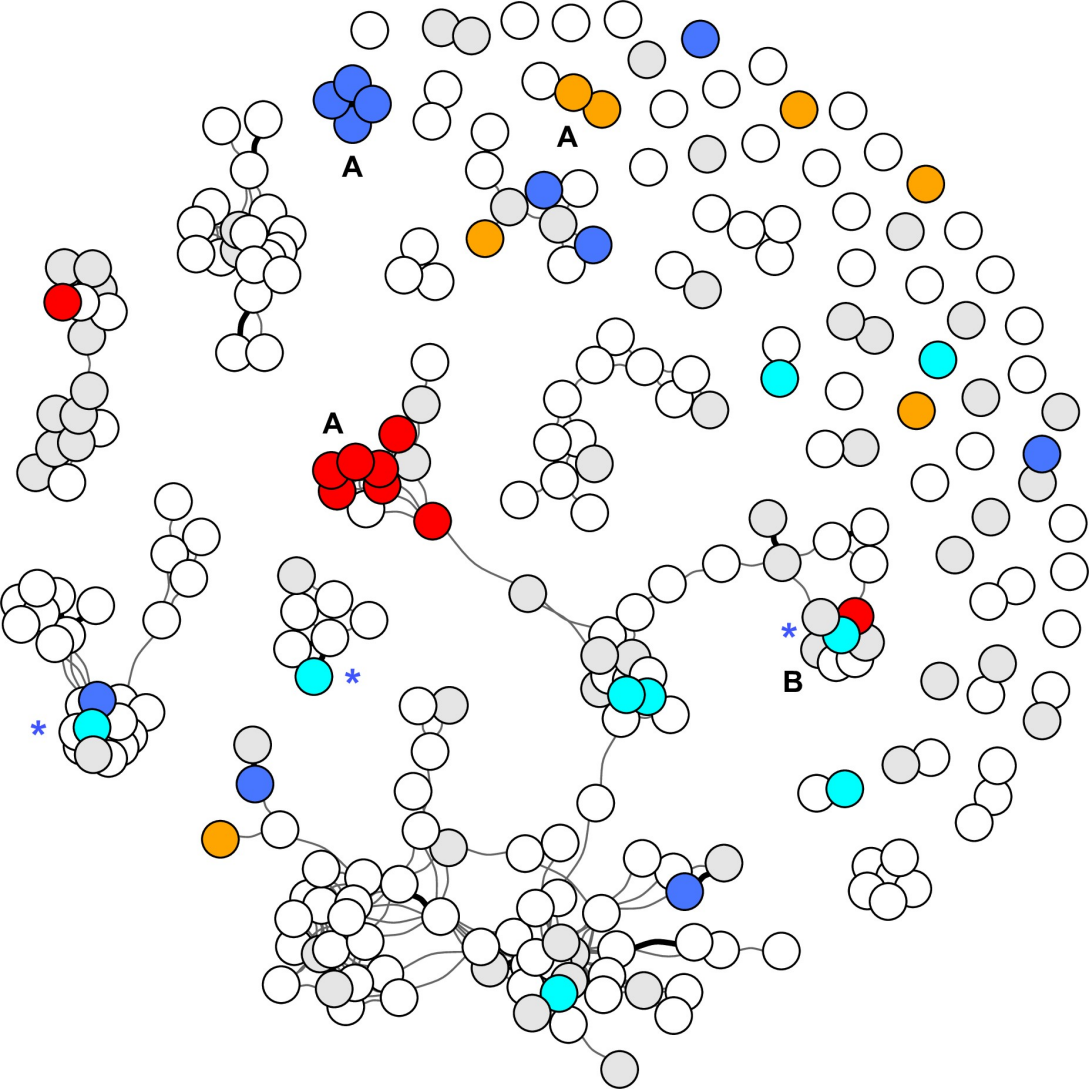
Social network of 275 birds diagnosed with avian mycobacteriosis. Each infected bird (n = 275) is represented as a circle (node) and all connections between them (edges) are shown for directly (dark line) and indirectly (light line) linked birds. Overlapping nodes tend to show clusters of highly connected groups of birds. For visualization purposes, the four most prevalent genotype groups, determined by comparison of whole genome sequences (WGS), are shown in colors. This included two groups of MAA (red, n = 9; orange, n = 7) and two groups of *M*. *genavense* (blue, n = 16*; turquoise, n = 9). Other birds with known genotypes are represented in gray (n = 59) and white circles indicate birds with missing WGS data (n = 178). Patterns of genotype groupings varied across the network. Similar genotypes clustered along paths of connectivity (e.g., A), dissimilar genotypes were found in connected birds (e.g., B), and some genotypes were dispersed throughout the network (e.g., orange, blue, and turquoise). Three birds with the turquoise genotype had a multiple infection with the blue genotype; these are shown in turquoise with a blue asterisk (*).

The social network consists of 275 nodes (one for each case) and the 461 edges that directly or indirectly connect them. Of these, 338 edges are between the eligible study subjects and their friends, while 157 edges directly connect cases to each other, totaling over 77,000 bird-days of direct case-case exposure. An additional 79 birds were linked to other cases indirectly. Thus, 86% (236/275) of all cases were directly- or indirectly-connected by one or two degrees of separation in this network. The four most prevalent genotype groupings, along with known and unknown genotypes are shown in color in Fig 1. Temporal and spatial clusters of both similar and dissimilar genotypes were visually observed, and some genotypes were dispersed throughout the network.

**Fig 2:**
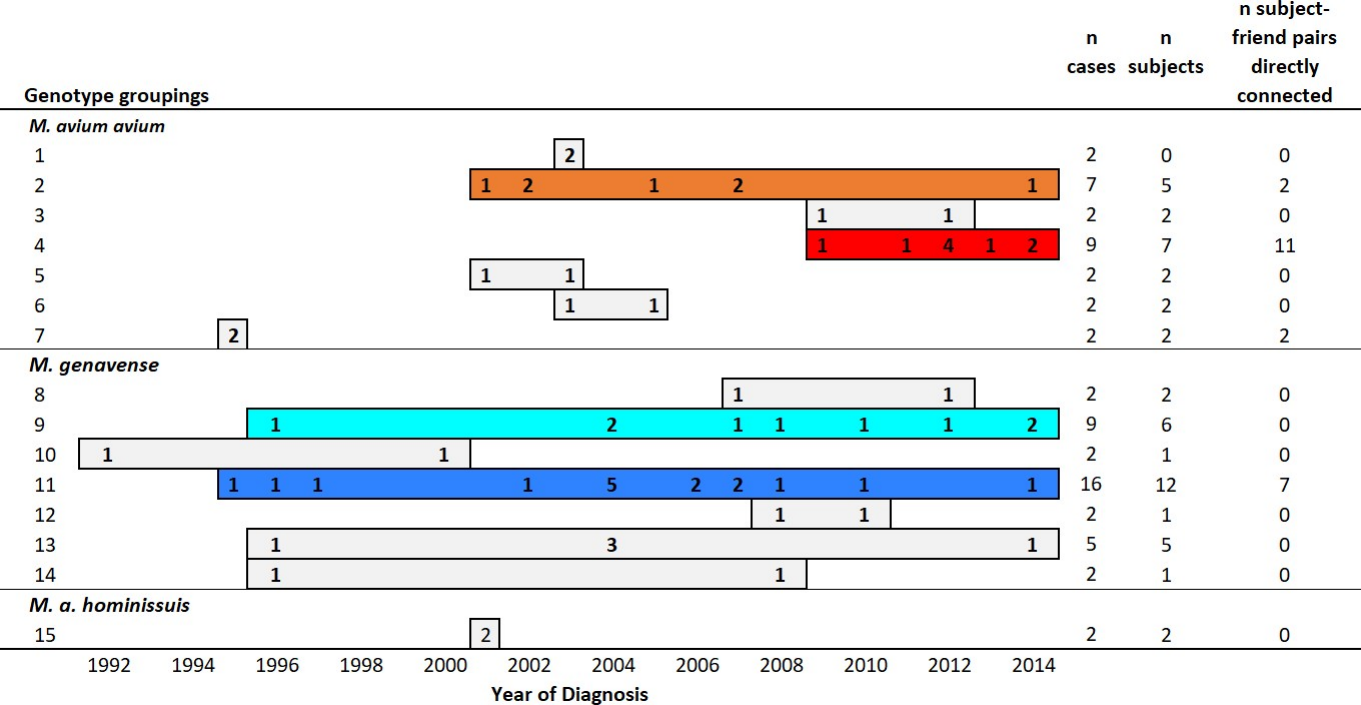
Case counts and temporal dynamics of the 15 groupings of similar genotypes that were identified in more than one bird. Each bar represents the time period over which that genotype group was detected in birds diagnosed with avian mycobacteriosis. Counts of cases diagnosed within any given year are provided. Colored bars identify genotype groups shown in Fig 1. Other shared genotypes are shown in gray, and non-shared genotypes are not shown. Additional details on birds with information linking them to phylogenetic analyses [20] are included in S1 File.

The 15-shared genotypes were detected over time (Fig 2). Most notably, similar *M*. *genavense* genotypes persisted in the population over long periods of 18 and 19 years (turquoise and blue genotype groups, Figs 1 and 2). Additional details are provided on birds with the 15-shared genotypes in S1 File, which includes isolate identification for linking specific genotypes to phylogenetic trees previously reported [20].

No differences in centrality measures in the network were identified between birds with MAA and *M*. *genavense* (Table 3). There was some evidence that birds with missing genotype data were more connected in the network than those where the genotypes were known (Kolmogorov-Smirnov p = 0.047 for eigenvector centrality; Table 3).

**Table 3:** Network centrality measures for 275 birds with avian mycobacteriosis at San Diego Zoo Global, 1992–2014.

	Centrality measures^a^					
	Degree centrality			Eigenvector centrality		
	median	IQR	p^b^	median	IQR	p^b^
All cases (n = 275)	14	(6, 43)		3.6x10^-12^	(1.7x10^-15^, 5.2x10^-10^)	
MAA (n = 41)	37	(12, 103)	0.65	1.3x10^-12^	(6.0x10^-16^, 4.7x10^-9^)	0.17
*M*. *genavense* (n = 44)	45	(13, 99)		1.3x10^-13^	(7.2x10^-16^, 1.6x10^-10^)	
Known genotype (n = 97)	43	(12, 110)	0.20	1.5x10^-13^	(6.5x10^-16^, 3.8x10^-10^)	0.047
Unknown genotype (n = 178)	26	(12, 75)		1.2x10^-11^	(4.8x10^-15^, 5.6x10^-10^)	

IQR = interquartile range; MAA = *Mycobacterium avium avium*

^a^Centrality measures were calculated from the entire bird network that consisted of 16,430 birds and 905,499 edges. Degree centrality = number of connected nodes; Eigenvector centrality = the extent to which a node is connected to other highly connected nodes.

^b^Kolmogorov-Smirnov p for the equality of distributions

#### Connected birds often had similar genotypes, especially among birds with MAA

Results of random network permutation tests are summarized in Table 4. Genetic data were available for subject-friend pairs in 61/338 pairs (18%). The proportion of these directly connected birds that had a similar genotype was 0.36 (22/61), and this was significantly different than random network permutations (p<0.001). The proportion with a similar genotype was higher when limited to the 26 pairs where both the subject and the friend had MAA (0.58; 15/26; p<0.001). The proportion was not significant among the subset of directly connected birds that both had *M*. *genavense* (0.29; 7/24; p = 0.25). None of the 11 birds with *M*. *a*. *hominissuis* were connected, and therefore network associations were not evaluated.

**Table 4:** Association between social network connectivity and genetic relatedness of mycobacteria among 275 cases of avian mycobacteriosis in birds at the San Diego Zoo and San Diego Zoo Safari Park (1993–2014).

Pairs of Cases	n connected pairs	observed proportion with similar genotype (n connected pairs with similar WGS genotype)^a^	Evaluation of genetic similarity	
			p-value (for proportion similar)^b^	p-value (for SNP distribution)^c^
**Directly connected**	338			
All pairs with WGS for both cases	61	0.36 (22)	<0.001	--
just pairs with MAA	26	0.58 (15)	<0.001	<0.001
just pairs with *M*. *genavense*	24	0.29 (7)	0.252	0.090
**Indirectly connected**	399			
All pairs with WGS that have the correct spatial and temporal alignment to test for a contagious process^d^	12	0.08 (1)	0.513	--
just pairs with MAA	8	0.13 (1)	--	0.014
just pairs with *M*. *genavense*	0	--	--	--

MAA = *Mycobacterium avium avium*, *’--’ =* not evaluated

^a^Genotypes between directly and indirectly connected cases were classified as “similar” (i.e., likely part of the same transmission chain) if they were within 12 SNPs of at least one other genotype in a phylogenetic group generated as previously described [19]; otherwise, they were characterized as "not similar".

^b^P-values were estimated from the null 95% confidence interval. If the observed proportion of connected birds with a similar genotype was outside the range of the 2.5th and 97.5th percentiles of the null distribution (i.e., the null 95% confidence interval), then the null hypothesis that the observed proportion could have arisen from chance was rejected.

^c^The Kolmogorov-Smirnov test for equality of distributions compared the observed distribution of SNPs between connected pairs to the distribution derived from 1000 random network permutations. Only pairs with MAA and *M*. *genavense* were evaluated, separately, in this comparison.

^d^Limited to case pairs where the friend of friend lived in a different enclosure than the subject, yet could have influenced the disease outcome in the subject based on the timing of contact with another mutual friend. This ‘friends of friends’ method [10] was used to isolate and test for patterns of contagion within the network structure.

The median number of days in-contact for directly-connected subjects and friends with MAA (n = 26) was 396 (IQR = 235–545; range 40–551). The length of time together did not differ between the MAA case pairs with similar (median = 397, n = 15) versus different (median = 413, n = 11) genotypes (Mann-Whitney U = 78, p = 0.78). For directly-connected *M*. *genavense* cases (n = 24), the median number of days in-contact was 158 (IQR: 92–348, range: 47–552). Days together also did not differ between *M*. *genavense*-connected cases with the same genotypes (median = 93, n = 7) versus different genotypes (median = 249, n = 17; Mann-Whitney U = 41, p = 0.25).

Data were sparse for evaluations between indirectly connected cases. Genotypes were known for 73/399 indirectly connected subjects and their friends of friends (18%); however, only 12 of these had the correct spatial and temporal alignment for evaluating hypotheses related to contagion [11]. Among these 12 indirectly connected case pairs, only one pair had a similar genotype (≤ 12 SNPs), and this was not significant (Table 4). Eight of these were MAA pairs (1 pair had ≤ 12; 7 pairs had > 12 SNPS), none were *M*. *genavense* pairs, and the other 4 were birds with different species of mycobacteria.

#### Distributions of SNPS support a contagious process for MAA that was not detected for *M*. *genavense*

When evaluating similarity of genotypes based on SNPs, isolates from directly connected cases of MAA (n = 26) were more similar than expected based on chance alone (i.e., fewer SNPs between connected cases in observed compared to random networks, p <0.001). Despite the small sample size, this pattern persisted among the indirectly connected MAA cases (n = 8; Kolmogorov–Smirnov test p = 0.014). On the contrary, no significant differences in SNP distributions between observed and random networks were detected among directly connected cases of *M*. *genavense*, perhaps because the number of pairs evaluated was small and, therefore, the statistical power was low. As previously stated, data were not available to evaluate SNP differences among the indirectly connected birds with *M*. *genavense*.

## Discussion

This is the first study to integrate mycobacterial WGS with a social network of birds and provides a new framework to investigate the epidemiology of avian mycobacteriosis. Our data included complete population identification, diagnostic information on all birds that died, and near-complete housing records for recreating exposure histories. Although genetic data were limited, the resolution of WGS with genome-wide comparisons is superior to conventional DNA fingerprinting for revealing true disease transmission dynamics [35, 36].

Mycobacterial species data were available for nearly half of the birds diagnosed with mycobacteriosis over the 22-year study period at SDZWA. In this large and fully enumerated population of diverse birds with post-mortem disease surveillance, 63% (78/124) of characterized Mycobacterium isolates were MAA or *M*. *genavense*. This finding is consistent with other reports [9, 13, 20, 37–39] which show these two species of Mycobacterium are the most prominent pathogens causing mycobacteriosis in birds. Therefore, understanding the transmission dynamics of these two species is an important consideration for managing avian population health.

We found greater genotypic similarity in isolates among cases that shared locational and temporal connections. This pattern was present when pooling data across all species of Mycobacterium and when limited to just birds with MAA. It was significant both when assigning plausible cutoffs for transmission events and when removing the cutoff assumption to examine genetic relatedness based on SNPs. While clustering of genotypes in directly connected birds would be expected for a contagious process, environmental point sources of infection could also produce genetic clusters. For example, similar WGS genotypes have been noted for *M*. *chimaera* outbreaks in hospitals resulting from a single environmental point source [40]. Among the small subgroup of case birds that were connected as friends of friends and never had contact with each other or each other’s enclosure, we found more genetic similarity, based on SNPs between connected birds with MAA than would be expected by chance. This means that, within this group, genetic similarities cannot be explained by contact to the same environment, leaving contagion as the main driver of pathogen relatedness [11]. This provides strong evidence that a contagious process is occurring among some MAA cases and is consistent with our previous analyses that did not incorporate genetic data [11].

For *M*. *genavense* we did not find evidence of disease clustering that would represent a contagious process, although the sample size was small. Using our genotyping method, *M*. *genavense* genotypes were very similar between connected and unconnected cases throughout the network. Temporal evaluations showed similar genotypes persisting over time (Fig 2), which may suggest propagation and maintenance of an infection through a population. It is possible that our limited number of cases combined with low genetic diversity [20] led to low statistical power for detecting a contagious process. This would be especially true if the social network was not optimized to capture specific timing and contact structure for transmission of *M*. *genavense*. It could also be that *M*. *genavense* is not as readily contagious as MAA. Others have suggested it has low pathogenicity due to lack of disease among in-contact birds [41, 42]. It is also possible that the environment is the primary source for *M*. *genavense* avian infections in the same way it is for human infections [43–45]. Differences in patterns between MAA and *M*. *genavense* may also reflect differences in host characteristics or sampling efforts. There was no evidence that birds with *M*. *genavense* and MAA had different opportunities to spread disease based on their location in the network (i.e., no difference in degree centrality or eigenvector centrality; Table 3). Additional studies clarifying transmission mechanisms and describing genetic diversity are needed to improve understanding of the epidemiology of *M*. *genavense* infections.

The measure of genetic similarity assumed that ≤ 12 SNPs was a sensitive and specific cutoff for identifying transmission events. This cutoff has been used as a threshold for ruling out transmission of *M*. *tuberculosis* between human hosts [28] and is based on low estimated base pair mutations rates of 0.3–0.5 SNPs per year [28, 36, 46]. There is evidence that MAA has a similarly low *in vitro* mutation rate of 1 SNP per genome per year [20]. Mutation rates have not been measured for *M*. *genavense*, but could be lower than other species of Mycobacterium based on the small genomic distance between all of our isolates [20]. Thus, it is possible that the ≤ 12 SNP cutoff does not correctly capture transmission dynamics for *M*. *genavense*. Our sparse data did not lend themselves to a robust sensitivity analyses of this cutoff that could be used to optimize a threshold for detecting transmission. To address the 12-SNP-assumption, we removed the cutoff and evaluated the distribution of SNPs in connected and non-connected birds. Both methods identified a non-random pattern of genetic similarity in birds with MAA that was not detected for birds with *M*. *genavense*. This suggests that our findings were robust to these different assumptions. Nonetheless, improved understanding of how mycobacterial diversity arises may better resolve transmission.

In total, 36% of connected cases had a similar genotype. If we assume that the sampled subset of birds is representative of all cases, then inferring that two clustered cases were caused by the same mycobacteria would have been wrong 64% of the time. These results show that even when an exposed bird becomes infected, it may not be from the same pathogen. Other studies have also documented case clusters that were eventually attributed to different mycobacteria using molecular methods [12–15]. Findings from the present study emphasize the need for improved avian mycobacteriosis screening and disease management protocols that address the high rate of false transmission observations. Recommended protocols have focused on breaking the bird-to-bird transmission through halted breeding, reduced movement in and out of exhibits, and depopulation [7, 47–51]. Improved methods that incorporate epidemiologic findings and genetic data into outbreak investigations could reduce the negative impact of disease management approaches on population breeding, sustainability, and reintroduction efforts.

Misclassification of network edges may explain some discordance between network connectivity and mycobacteria genomic data. Connectivity between subjects and friends was based on defining precise time periods when bacteria could accumulate from another shedding bird; however, enclosure sharing is only a proxy for true contact that would lead to disease transmission. Additionally, the time periods may not have captured potentially long periods of mycobacteria viability [52, 53] that could present a transmission risk after a shedding enclosure mate is removed. This could have misclassified some birds as not being connected, when they had a true epidemiologic link. We used historical reports [8, 21–23] and our own data [9] to estimate the incubation and infectious periods, but the true distributions of these important periods are unknown; however, sensitivity analyses from our previous study [11] showed no major differences in patterns of contagion when the risk periods were modified. For the specific pair-based genetic analyses with our small sample size, misclassification would weaken our ability to detect associations consistent with contagion.

We generated the largest, most comprehensive transmission network (based on high-resolution genetic data) ever reported for mycobacterial disease in birds. Nevertheless, genetic data were available for only about one-third of the cases, which translates into missing data for many of the subject-friend pairs. We may also have an incomplete inventory of genotypes among birds with isolates, which can complicate epidemiologic interpretations. Infection with multiple mycobacteria has been documented in this bird population [20], in other birds [15], and in humans [54–56]. Acquiring WGS data for cases was challenging, as it required culture of slow-growing, fastidious organisms that do not always culture well. There also had to be enough mycobacterial DNA in the sample to obtain high read coverage, which could limit the detection of multiple organisms, if present [20]. Despite the limited data, there were enough pairs of cases with WGS to test for network effects for MAA and *M*. *genavense*; however, there were not enough cases with *M*. *a*. *hominissuis* to evaluate patterns of genetic similarity (i.e., only one pair had similar genotypes, and no cases were connected). Following this cohort of birds into the future to obtain additional mycobacterial WGS may fill data gaps.

The significant differences in proportions of MAA or *M*. *genavense* isolates from Anseriformes, Columbiformes, and Passeriformes suggest that certain mycobacterial species may be more common in some avian taxa. These findings may represent true differences in bird species’ susceptibility to the different mycobacteria or highlight varying exposure of birds with different life history traits to mycobacteria from different ecological niches.

Environmental reservoirs of *M*. *avium* have been extensively reviewed [17, 57] given this pathogen’s role in non-tuberculous mycobacterial infections in humans worldwide. Along with reports of *M avium* in soil, food, and plants [57], this mycobacterium has notable hydrophobic properties that allow it to adhere to pipes and congregate in biofilms [58, 59]. *M*. *avium and M*. *avium* complex infections in human patients have been traced to specific environmental sources in several studies, including a hospital water supply [60], biofilm in a showerhead [61], general household plumbing [62], and hot tubs [63, 64]. The strong affiliation of MAA with water and aerosolization of mycobacteria as a route of infection may explain our finding for disproportionately high numbers of MAA infections in Anseriformes, compared to other taxa, and is consistent with other reports of mycobacteriosis due to *M*. *avium* in large groups of waterfowl [39, 65, 66].

*M*. *genavense*, on the other hand, was not present in any of the Anseriformes, but was identified in 79% of the passerine cases (perching birds), which spend comparatively more time in arboreal habitats. Less is known about the natural habitat and reservoirs of *M*. *genavense*, although it has been found in tap water [44] and is generally described as having environmental or avian reservoirs [44], where it has commonly been identified in Psittacines and other pet birds as well as various zoo birds [37, 38, 41, 67].

The disproportionality of infection from the different pathogens across avian taxa offer new avenues of research that can reveal infection routes and inform disease control efforts. Importantly, our results assume that the birds with mycobacterial isolates (Table 2) are an unbiased subset of all cases; further studies to obtain WGS from mycobacterial isolates representing more species of birds are ongoing and may provide new insights on disease epidemiology.

Social network analysis coupled with traditional epidemiologic contact tracing and whole genome sequencing helps refine our understanding of disease epidemiology. Our results show that some, but not all, spatial and temporal clusters of cases were genetically similar. Significant patterns of genetic relatedness between friends and between friends of friends strongly suggest a contagious process is occurring in some situations. By contrast, clusters of cases with genetically unrelated mycobacteria suggest that infection may arise from independent sources or from transmission pathways that have not been completely elucidated. Our findings provide new insights into the complex disease epidemiology and suggest that avian mycobacteriosis is not a single, homogeneous disease entity and that drivers of disease may differ for MAA and *M*. *genavense*. These insights can better inform disease control strategies in zoos and other managed populations.

## Supporting information

S1 File Information on birds with shared genotypes and their genetic clusters separated by 12 or fewer SNPs, as reported by Pfeiffer et al., 2017.^a^ (XLSX)
